# Systemic therapy of necrobiotic xanthogranuloma: a systematic review

**DOI:** 10.1186/s13023-022-02291-z

**Published:** 2022-03-24

**Authors:** Lisa Steinhelfer, Thomas Kühnel, Herbert Jägle, Stephanie Mayer, Sigrid Karrer, Frank Haubner, Stephan Schreml

**Affiliations:** 1 Department of Dermatology, University Medical Center Regensburg, Franz-Josef-Strauß-Allee 11, 93053 Regensburg, Germany; 2 Department of Nuclear Medicine, Technical University Munich, Ismaninger Strasse 22, 81675 Munich, Germany; 3 Department of Radiology, Technical University Munich, Ismaninger Strasse 22, 81675 Munich, Germany; 4 Department of Otorhinolaryngology, University Medical Center Regensburg, Franz-Josef-Strauß-Allee 11, 93053 Regensburg, Germany; 5 Department of Ophthalmology, University Medical Center Regensburg, Franz-Josef-Strauß-Allee 11, 93053 Regensburg, Germany; 6 Department of Internal Medicine III, University Medical Center Regensburg, Franz-Josef-Strauß-Allee 11, 93053 Regensburg, Germany; 7 Department of Otorhinolaryngology, Ludwig Maximilians University Munich, Marchioninistr. 15, 81377 Munich, Germany

**Keywords:** Necrobiotic xanthogranuloma, Non-Langerhans cell histiocytosis, Systemic therapy, Necrobiotic xanthogranuloma and therapy

## Abstract

**Background:**

Even though a plethora of systemic therapies have been proposed for necrobiotic xanthogranuloma (NXG), there is no systematic review on this topic in literature.

**Objective:**

To review all existing literature on the systemic therapy of NXG in order to identify the most effective therapies.

**Methods:**

All reported papers in the literature were screened for systemic treatments of NXG. Papers without proper description of the therapies, papers describing topical therapy, and articles without assessment of effectiveness were excluded. Subsequently, we analyzed 79 papers and a total of 175 cases.

**Results:**

The most effective treatments for NXG are intravenous immunoglobulins (IVIG), corticosteroids, and combination therapies including corticosteroids.

**Conclusions:**

Corticosteroids and IVIG should therefore be considered first-line treatments in patients with NXG.

**Supplementary Information:**

The online version contains supplementary material available at 10.1186/s13023-022-02291-z.

## Background

Necrobiotic xanthogranuloma (NXG) was first described by Kossard and Winkelmann in 1980 and is a rare non-Langerhans cell histiocytosis with no gender preference. The disease mostly affects patients in the sixth decade of life and is associated with cell proliferative disorders, such as multiple myeloma (MM) or monoclonal gammopathy of undetermined significance (MGUS). The etiopathogenesis of necrobiotic xanthogranuloma is unknown. However, It is conceivable that paraproteins play a role as a trigger or cofactor for granuloma formation [1–4] (more background information in Additional file 1). NXG often initially presents with yellowish or brownish macules and nodules. As the disease progresses, atrophies, telangiectasias, ulcerations and scars may be present within the lesions [5]. The lesions are usually asymptomatic and often appear in the periorbital area. In a few cases, systemic involvement was found in autopsies [6–8]. The most common extracutaneous localizations comprise the oropharyngeal tract, the bronchi, liver, spleen, lung and heart [9–13] Histopathologically, NXG is characterized by granulomas in the dermis extending into the subcutaneous fat. Atypical foreign body giant cells of the Touton type are often found [14]. Cholesterol clefts are a hallmark of the disease [15] (also see Additional file 1). Due to the rarity of NXG, mostly case reports and case series exist. A lot of patients with NXG will receive several drugs before getting proper treatment.

## Materials and methods

### Eligibility criteria

Studies were included when patients were at least 18 years old and diagnosis was histologically confirmed. We screened cohort studies, case–control studies, case series, case reports and letters that clearly reported the outcome of the respective systemic treatments. As we focused on systematic therapies, papers dealing with topical treatments were excluded. In addition, some articles were removed due to duplicate information. Studies were checked for eligibility by the first author, and then results were reviewed by the last author.

### Information sources/study selection

A review by Miguel et al. helped to identify relevant cases from 1980 to 2014. Only patients who had received systemic therapy were included. As a second step, we searched PubMed, Medline and Web of Science databases using the queries “necrobiotic xanthogranuloma and therapy” until 2021. Following the database search, studies were compiled into a single list with all duplicates removed. Further exclusion criteria were studies with aggregated data, an unclear diagnosis, only topical treatment mentioned, no proper description of treatment, or response to treatment not mentioned.

### Outcome assessment

The primary outcome was the reported response to systemic treatment in the papers. These were classified as “complete response”, “partial response”, “stable disease” or “progressive disease”. The response to therapy was evaluated by reviewing each patient’s medical record (as reported). Complete response to treatment was used for the absence of all detectable NXG lesions and stable hematological symptoms. Partial response was defined as a decrease in the size or number of NXG lesions and an improvement of the hematological symptoms. Stable disease was defined as no change in the size or number of the NXG lesions and stable hematological symptoms. Progressive disease was defined as an increase in the size or number of the NXG lesions or worsening of the hematological condition. In mixed response scenarios (reduction in size or regression of individual lesions with simultaneous appearance of new lesions), we rated as “progressive disease”. The sole response of cutaneous lesions with simultaneous progression of the hematological condition, or vice versa, were also rated as “progressive disease”.

## Results

### Study identification

**Fig. 1:**
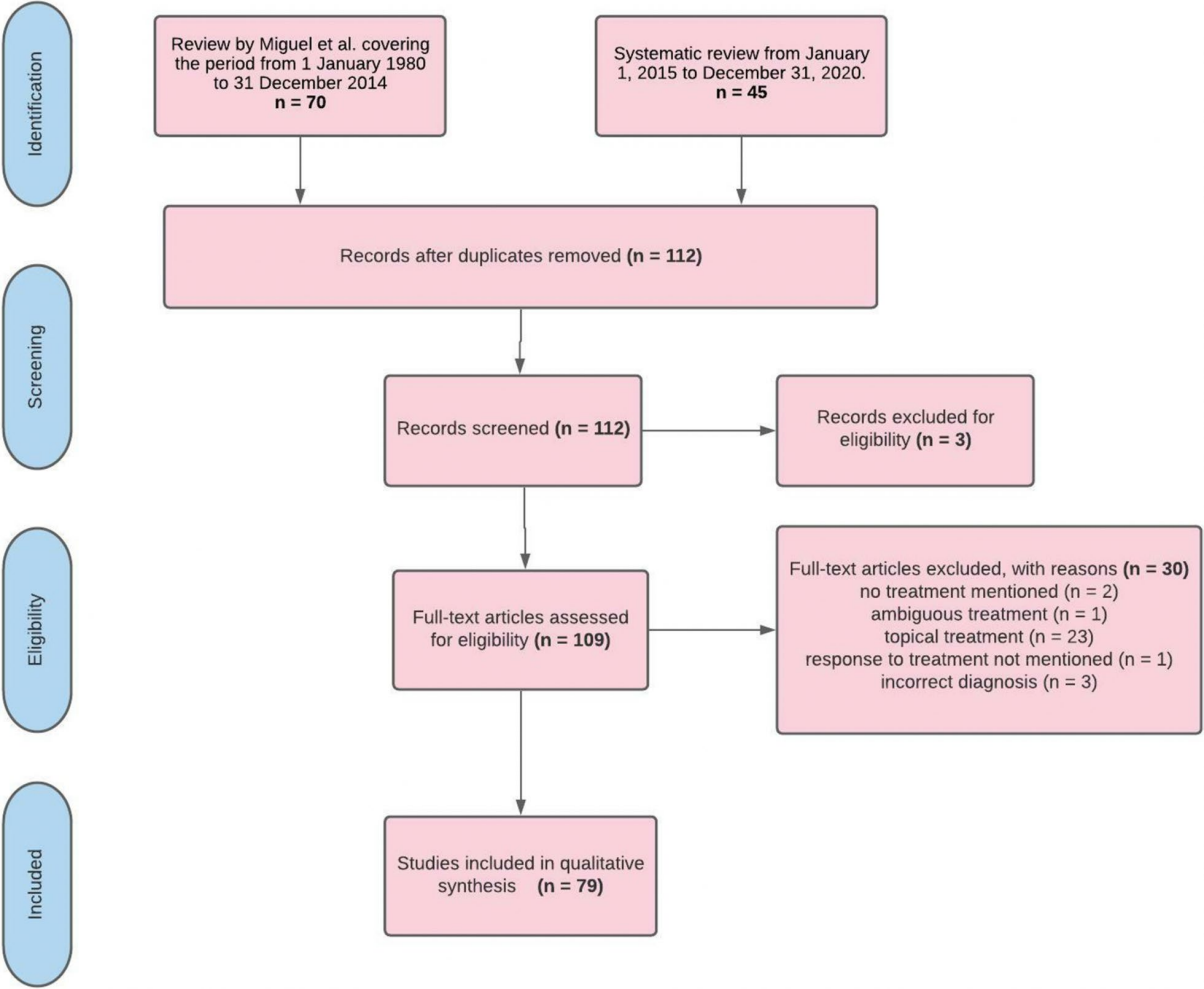
PRISMA flowchart of the study. The selection process for study inclusion in the systematic review and meta-analysis according to the preferred reporting items for systematic reviews and meta-analysis. A total of 170 patients were included from the literature search. 5 more institutional cases were added (see Table 1, Additional file 1, and Fig. 2)

**Fig. 2:**
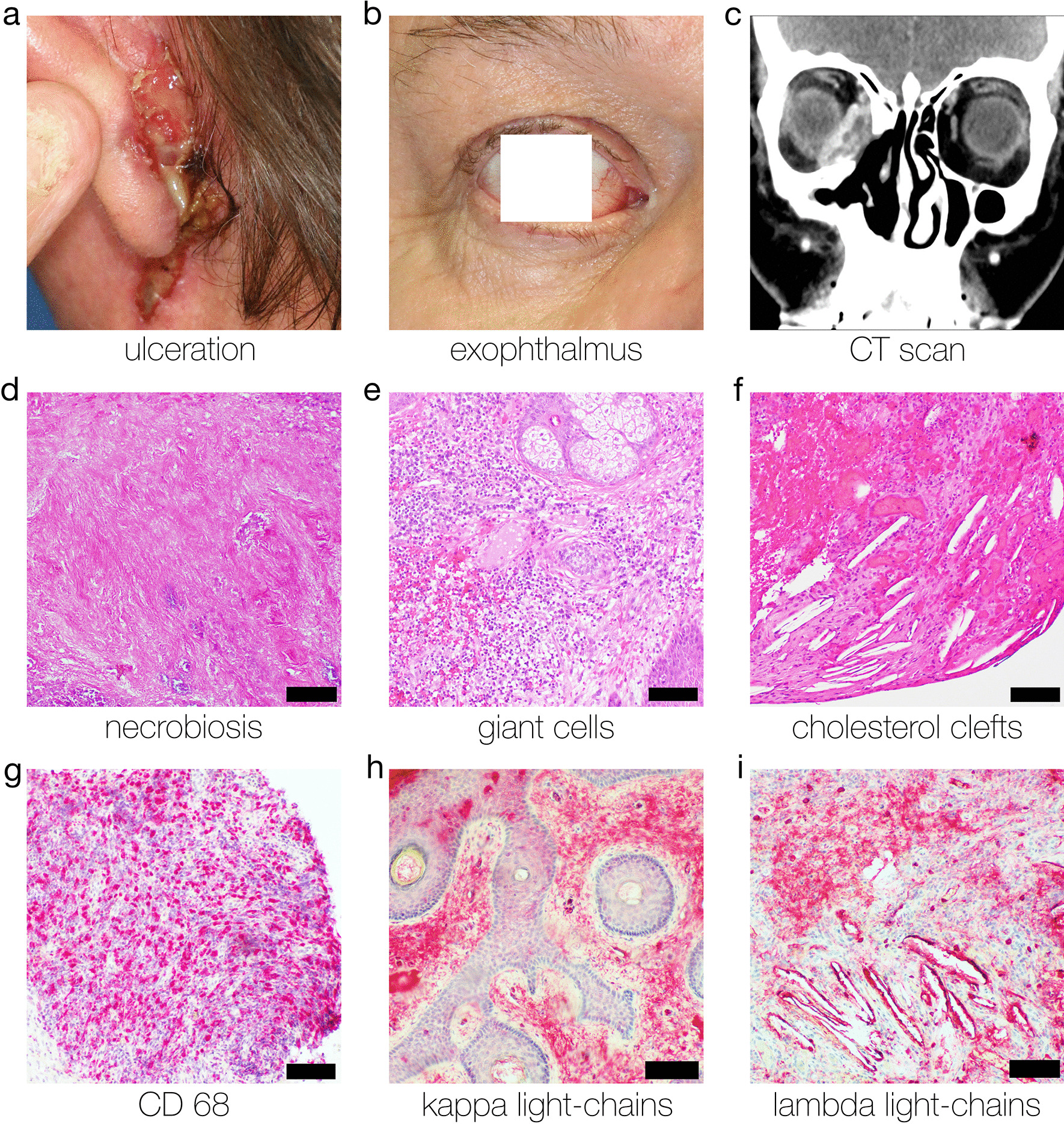
Institutional case 2. Clinical findings, CT scans, histological features and immunohistochemistry. Also see Additional file 1 and Table 1. Scale bars, 200 μm

The review by Miguel et al. helped to identify 101 patients [1–3, 14–59]. The additional literature search yielded 45 records. After removal of duplicates, 39 papers were subject to fulltext-review. 13 records were excluded: 6 did not discuss systemic treatment of NXG, a further 2 did not report any treatment, another study provided ambiguous information on treatment, 3 studies discussed an alternative diagnosis to NXG and another study failed to mention the response to treatment. A total of 26 studies were included based on the above-mentioned criteria. These 26 articles present the therapy options and the course of therapy of 69 patients [4, 60–84]. 5 institutional patients (University Medical Center Regensburg) were included (Table 1, see Additional file 1). We were thus able to assess the outcome of systemic therapies in 79 studies and 175 patients (Fig. 1, representative institutional case in Fig. 2).

**Table 1:** Clinical data of institutional case reports: For details, see Additional file 1

Case	Age	Cutaneous involvement	Organ involvement (extracutaneous)	Prior treatments	Latest treatment	Response	Follow up
1	59	Extensive ulcerations on both sides of the lower legs with erythematous wound edges	Whitish discoloration of right cornea, left eye sunken back into the orbita, purulent discharge bilaterally	None	Oral corticosteroids (prednisolone)	Partial response	3 months later her condition had deteriorated
2	61	Ulceration with destruction of the auricular lobe on the left ear	Double vision and visual disturbances (due to infiltration of the eye muscles)	(a) Intravenous immunoglobulins (IVIG) (b) Cytoreductive therapy with melphalan	Chlorambucil and oral corticosteroids (prednisolone)	Partial response	> 12 months progression free (survival)
3	72	Generalized cutaneous and subcutaneous lesions	Osseous involvement, mediastinal and hilar lymph node involvement on both sides, bilateral involvement of the lung	(a) Cytoreductive therapy with melphalan (b) lenalidomide in combination with dexamethasone	Dapsone and oral corticosteroids (prednisolone)	Partial repsonse	> 12 months progression free (survival)
4	69	Ulceration of the chest, the neck and the perorbital region	Vestibular and cochlear involvement, olfactory system was also involved	Lenalidomide (stopped because of pronounced leukocytosis)	Lenalidomide (reduced dose of 5 mg/day) and oral corticosteroids (prednisolone)	Stable disease	3 months later her condition had deteriorated
5	67	Bilateral yellowish swelling of both upper and lower eyelid	None	None	Oral corticosteroids (dexamethasone, tapered slowly to Cushing threshold)	Partial response	> 12 months progression free (survival)

Images from Case 2 are given in Fig. 2

### Bias and quality assessment

Most of the studies were case reports and some were case series and the sample size of all studies was small. Since a scale for severity of NXG does not exist, clinical response is difficult to classify. In our systematic review, clinical response is essentially based on the individual report of each author. This makes a comparative statements difficult, which is a limitation of the study. All studies were uncontrolled, leading to a high risk of confounding. Due to the lack of randomization, the risk of selection bias was high. Risk of reporting bias was high due to lack of blinding. It is difficult to comment on publication bias, however, as the main part of evidence is from case reports, the question arises whether ineffective therapies have been published in the same way as effective ones.

### Patient demographics

The most common association between NXG and hematologic disorders has been with plasma cell dyscrasias. Of the 175 patients, 95 patients had paraproteineinemia (55%). The most common subtype was IgG-kappa. 19 of 175 (11%) patients had a malignant condition: Multiple myeloma, in 12 patients (7%), was the most common type. However, Hodgkin lymphoma and chronic lymphatic leukemia (CLL) were also observed. The overall perecentage of patients with simultaneous paraproteinemia and/or a malignant condition was 65% (114 patients).

### Systemic therapies

Different treatments have been used for NXG with a wide variety of responses, such corticosteroids, IVIG, lenalidomide, cyclophosphamide, chlorambucil, thalidomide, melphalan, infliximab/rituximab, cladribine, bortezomib, vincristine, interferon alpha-2a, dapsone, ibarubicin, adalimumab, etretinate, cyclosporine, mycophenolate-mofetil, clofazimine, minocycline, doxycycline, acitretin, azathioprine and combined therapies (FCR, RCVP, vincristine/melphalane/cyclophosphamide/prednisolone).

### Effect of interventions

**Fig. 3:**
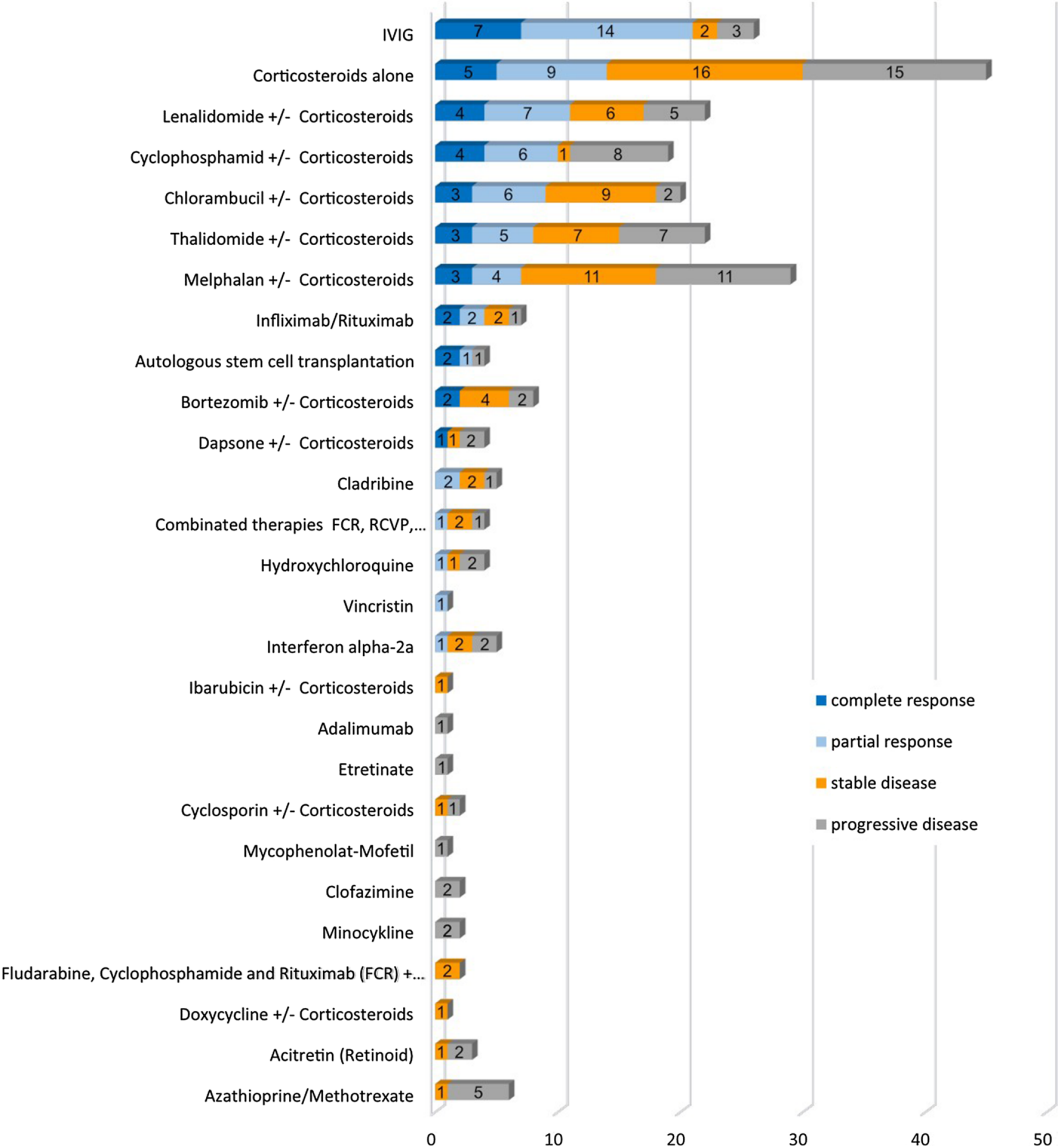
Efficacy of systemic therapies for necrobiotic xanthogranuloma. The numbers denote the cases with respective treatments

The effect of treatments administered are presented in Fig. 3. Corticosteroids were the most frequently used treatment for NXG. Corticosteroids were used in 45 cases. Complete response occured in 5 patients (11%), and partial response in 9 patients (20%), stable disease was achieved in 16 patients (36%) and progressive disease was observed in 15 patients (33%). The use of IVIG turned out to be the most effective therapy. IVIG were used in 26 patients. Complete response was achieved in 7 patients (27%) and partial response in 14 patients (54%). Two patients exhibited stable disease (8%) and three patients did not respond to the therapy (11%). Another sufficient therapy option was the use of lenalidomide in combination or without corticosteroids. Complete response was observed in 4 patients (18%) and partial response in 7 patients (32%). Six patients (27%) achieved partial response and no response was noticed in five patients (23%).

The overall response was improvement (complete response and partial response) in 128 patients (73%) and stable disease in 25 patients. 22 (13%) patients showed progress of disease.

Since patient data were collected from individual case reports, follow-up data were only occasionally available. The duration of response was set to be at least the timespan reported in the case reports in case patients were either lost to follow-up or no other information was given.

Of the 26 patients treated with IVIG, follow-up data were available for 8 patients. The median duration of response (2 patients with complete response and 6 patients with partial response) for the 8 patients was 12 months (range 6–48 months, mean 15.75 months). Furthermore, we wanted to illustrate the follow-up data of the second promising therapy, the use of corticosteroids. Of the 45 patients treated with corticosteroids, follow-up data were available for 10 patients. The median duration of response (4 patients with complete response, 5 patients with partial response and 1 patients with stable disease) for the 10 patients was 12 months (range 2–24 months, mean 11.9 months).

## Discussion

This study provides a systematic review on the systemic treatment of NXG. IVIG had the best response rate (21 of 26 patients [81%] with complete or partial response), followed by corticosteroids (30 of 45 patients [67%] showed response or stable disease), and lenalidomide in combination, or without corticosteroids (17 of 22 patients [77%]). However, other therapeutic agents were frequently used in combination therapies. It is challenging to draw conclusions regarding the effectiveness of combination treatments due to the low number of reports for each combination. Furthermore, it is difficult to evaluate the response to therapy as there is no standard rating scale for the severity of NXG. The clinical response or results are based on each author’s individual report. In conclusion, despite the notable limitations of the currently available data (case reports, rating system could be varied, interpretation of case report data), this systematic review suggests that the therapeutic use of IVIG and corticosteroids are the most promising drugs to achieve disease control in NXG. As there are still no clear guidelines in the therapy of NXG, prospective, comparative, randomized controlled trials would be required to determine the best therapeutic approach. However, this will hardly be feasible due to the low number of cases.

## Conclusions

Our study shows that the most effective treatments for NXG are intravenous immunoglobulins (IVIG), corticosteroids, and combination therapies including corticosteroids. Therefore corticosteroids and IVIG should be first-line treatments in patients with NXG.

## Supplementary Information

**Additional file 1**. Supplementary information.

## Data Availability

Available upon reasonable request.
