# Supplementary material for: Systemic therapy of necrobiotic xanthogranuloma: a systematic review
Source: Orphanet J Rare Dis. 2022 Mar 24;17:132. doi: 10.1186/s13023-022-02291-z (PMC8944121; doi:10.1186/s13023-022-02291-z)
Supplement: Supplementary file 1 — Additional file 1. Supplementary information. [file 13023_2022_2291_MOESM1_ESM.pdf]

Supplementary information

Periorbital distribution

NXG often presents with peri- or intraorbital involvement, that may lead to vision changes, diplopia, episcleritis, keratitis, iritis, conjunctivitis, corneal perforation. Enlargement of the lacrimal glands and involvement of the extraocular muscles and fat and can also be present. Despite the fact that the periorbital region is affected in about 80% of the patients, the presence of periorbital involvement is not a pathognomonic criterion [1-3]. The diagnosis of NXG with periorbital manifestation has to be confirmed via histology [4].

Hematologic disorders associated with NXG

Most patients (80%) with NXG have paraproteinemia, which is usually of the IgG kappa type, but only 10% of patients develop multiple myeloma. Lymphoproliferative B-cell diseases, such as B-cell chronic lymphocytic leukemia, are also associated with NXG. There have also been reports of associated cryoglobulinaemia, anaemia, leucopenia, hypocomplementaemia, hyperlipidaemia, impaired glucose tolerance and elevated erythrocyte sedimentation rate [5]. Serum cholesterol and triglyceride levels do not correlate with the disease and are normal in most cases. Hematologic disorders may arise before or after the onset of skin lesions. Therefore, patients with NXG require lifelong follow-up care[6].

Histological findings

NXG has several features histopathological features that are often present. Such as extensive zones of degenerated collagen surrounded by xanthomatous histiocytes in a palisaded arrangement within the dermis. Cholesterol cracks are also observed. Histiocytes can be both of the epithelioid and the foam type [7]. A vacuolated cytoplasm and nuclei with light chromatin and a small nucleolus can be detected microscopically [8]. Even with low microscopic magnification, multinucleated giant cells of the Touton, Langerhans and foreign body type were prominent. In immunohistichemistry, CD68, CD163 and MAC387 positive cells are present. In contrast, they are negative for CD1a and S100 [9] [10].

Systemic involvement

NXG can also affect multiple organs. Umbert and Winkelmann reported a patient, who had xanthogranulomas on the back, flanks and forehead[11]. The histopathological examination of a skin biopsy revealed characteristics of NXG. The chest X-ray showed linear fibrosis in both lower lobes with calcified granulomas in the right lower lobe [11]. Hunter and Burry described myocardial lesions in NXG patients for the first time as part of an autopsy[12]. In addition, they reported an autopsy of a 39-year-old woman with NXG, who had myeloma with paraproteinemia. The larynx, pharynx and kidneys were also affected by necrobiotic lesions. Nodular changes in the liver were also found, which led to portal hypertension [12]. In 1952, Frank and Weidman described a patient with skin lesions as well as an involvement of muscle and myocardium [13]. An outstanding feature in the case report of Nowak et al. was the affection of the left ventricle and nodular transformation of the liver by NXG[14]. The autopsy revealed extensive nodules, plaques, scars and ulcers of the abdomen, arms and legs. The heart was slightly enlarged with concentric left ventricular hypertrophy, yet showed functioning valves. The patient had three yellow-whitish, plaque-like necrotic areas in the myocardium of the left ventricle, subepicardially and interventricularly. Hepatic and splenic granulomas have also been found in patients [14]. There are some reports on the occurrence of NXG within scars, being present within linear morphea [7]. Winkelmann et al. reported lesions on a chest mediastinoscopy scar. In their report, in addition to the scar other skin areas were affected, such as the scalp and lower eyelid [15].

Institutional case reports

Case 1

Figure S1

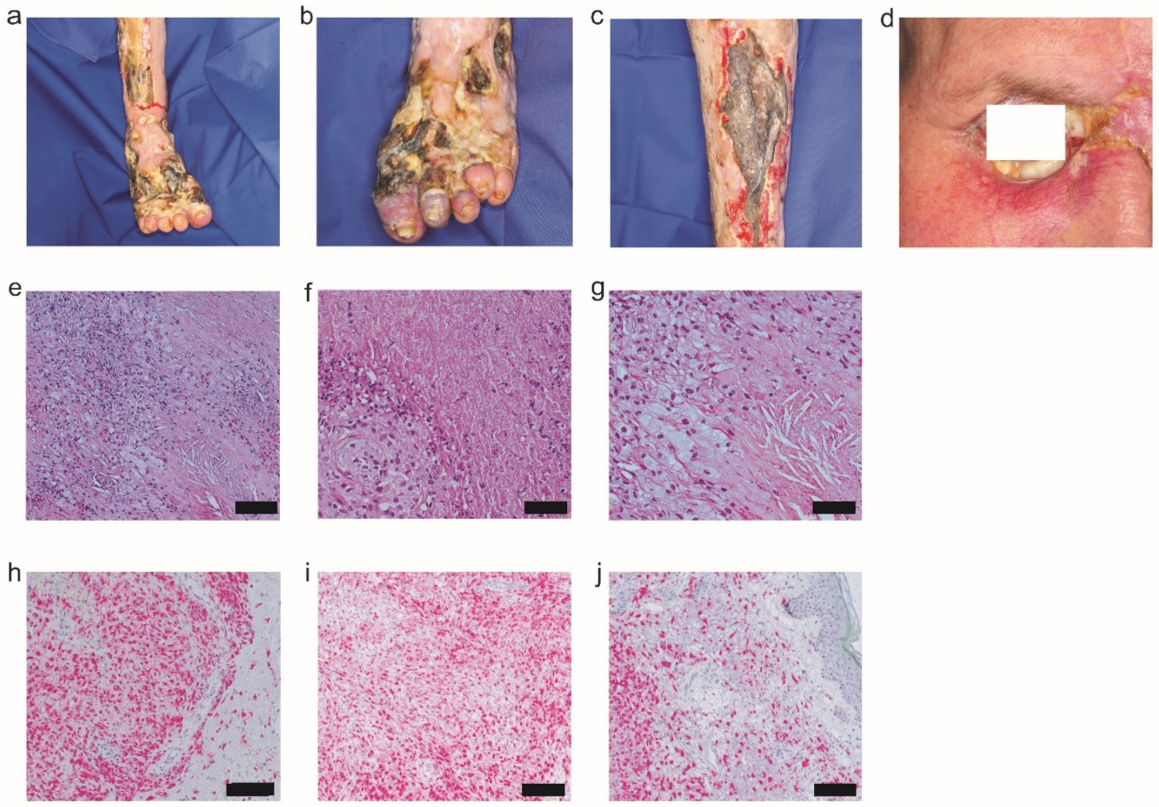

Figure S1: A-D, clinical images; E-G, histology (H&E, bar = 200 µm) showing necrobiosis (e,f), cholesterol clefts (g) and foamy histiocytes (e-g); H-J, immunohistochemistry for CD68.

Clinical presentation

A 59-year-old woman presented with a 7-year history of gradually enlarging extensive ulcerations on the lower legs and forefeet on both sides (Fig. X). By the time of admission she had extensive smelly ulcerations on both sides of the lower legs with fibrinous deposits and erythematous wound edges. Deep black necrotic areas measuring 20x6cm on the front edge of the tibia. On the right upper arm there was an approximately 1.8 cm large, slightly scaling lesion with crusty deposits and reddening of the surrounding area. The cornea on the right eye was discolored whitish. The left eye had fallen back into the orbit and purulent outflow was draining

on both sides. Venous malformations, Triple-Trenaunay syndrome and a chronic pain syndrome were known to be secondary diagnoses. In addition, there was anemia, thrombocytosis and hypoalbuminemia.

Histological findings

Histological examination showed extensive dense infiltrate of histiocytic cells with large, foamy cytoplasm and large cells. The entire corium is filled with a mixed-cell inflammatory infiltrate, which shows a clear granulomatous aspect in the edge area of the excidate. Occasional foreign body giant cells could be found. No cholesterol clefts could be detected. CD3 and a CD68 stain were also carried out. The infiltrate cells were strongly CD68-positive. In the CD3 staining, a few lymphocytes were detectable in the subepidermal and basal areas.

Therapy

We treated her with high-dose oral prednisolone. She was given 80mg daily. As a co-medication, the patient received osteoporosis and candidiasis prophylaxis. The patient received regular Prontosan compresses for local disinfection of the ulcerations on both lower legs and forefoots.

Outcome

She had an encouraging response to this treatment, the purulent secretion from both orbits decreased. The ulcer environment gradually faded. The patient also reported decreasing pain. The local findings showed overall good response to the therapy with corticosteroids. The medication was slowly reduced to prednisolone 40 mg. Although there had been significant clinical improvement initially, at review 3 month later her condition had deteriorated.

Case 2

Figure S2

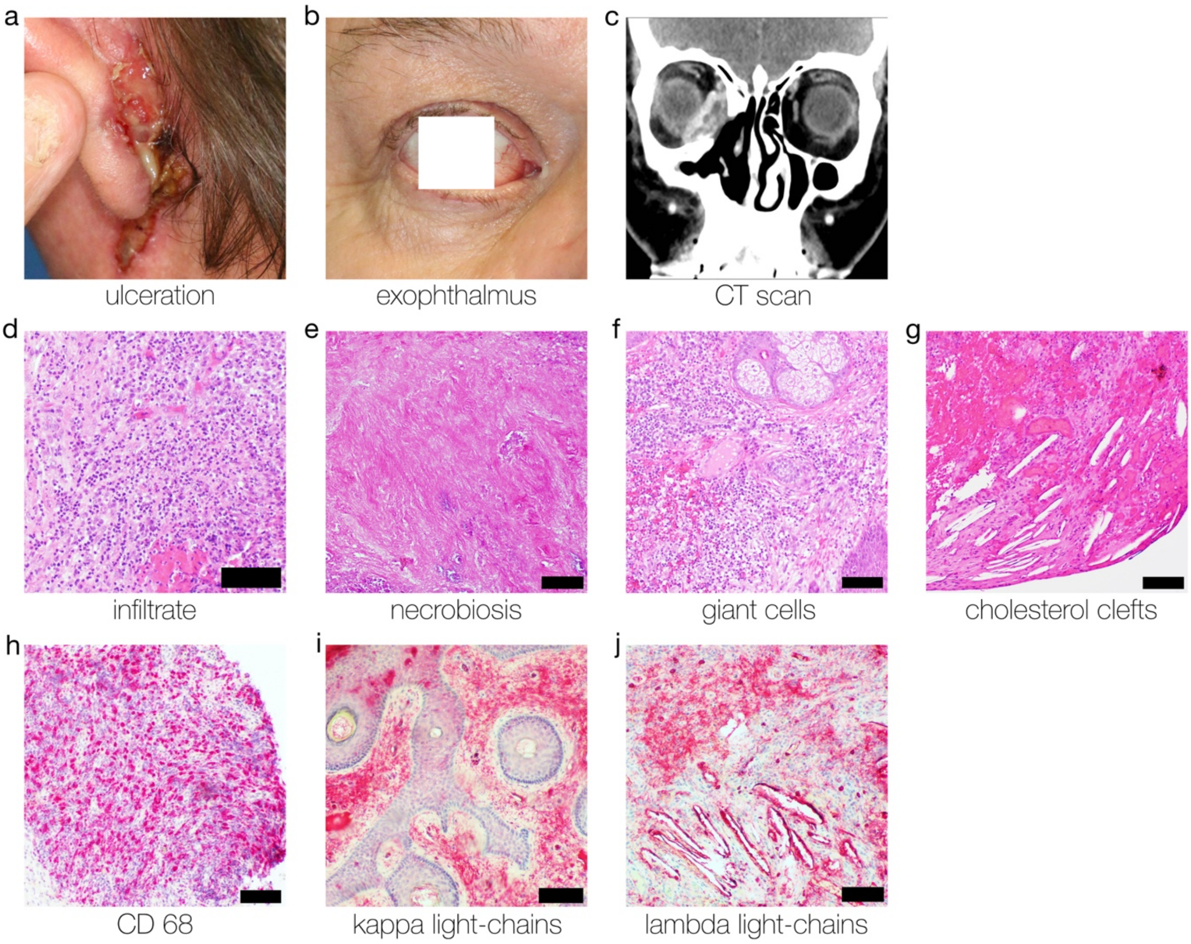

Figure S2: A/B, clinical images; C, CT scan, D-G, histology (H&E, bar = 200 µm) with inflammatory infiltrate, necrobiosis, multinucleated giant cells and cholesterol clefts; H-J, immunohistochemistry for CD68 and immunoglobulin light chains.

Clinical Presentation

A 61-year-old man suffered double vision and visual disturbances for approximately two months.

A tissue sample that had previously been taken from the eye was re-examined by us. A biopsy was performed on the right eye revealed the diagnosis of necrobiotic xanthogranuloma. A substance defect with destruction of the lobe auriculae was found on the left ear.

Histological findings

Histological examination showed extensive area of necrobiosis and numerous foamy histiocytes. In addition, it was possible to delineate many plasma cells and some cholesterol columns. The immunohistochemical staining showed clear positivity of the histiocytes for CD68.

Therapy

Treatment was started with intravenous immunoglobulin (IVIG). This was given to the patient in five single doses for a total of five days. We supported the therapy with Calcilac chewable tablets for osteoporosis prophylaxis, pantoprazole 40mg daily for stomach protection and Ampho Moronal Suspension four times a day for candida prophylaxis. After three cycles of immuninglobulin therapy, evaluation was carried out using cCT showing the disease was progressing. Therapy with prednisolone 40 mg once a day was initiated. And the patient was admitted to the hospital to start cytoreductive therapy with melphalan using 2 mg daily. In addition, a prompt surgical resection of the intraorbital tumor was recommended. Five weeks after resection of the necrobiotic xanthogranoloma in the area of the right orbit the patient reported that he was in good general condition, but double vision have occurred more frequently. Eye motility was also increasingly restricted. Since the therapy with melphalan was paused postoperatively, there has been increased oozing in the area of the right ear and lacrimation in the area of the right eye. The MRI examination of the skull showed a clear progression of necrobiotic xanthogranuloma in the right orbiata. We decided to try another cytoreductive therapy, this time with chlorambucil and prednisolone (Chlorambucil 4 mg once a day orally in combination with prednisolone 10 mg once a day).

Outcome

After treatment with chlorambucil and corticosteroids the patient noticed improvement in double vision and visual disturbances and almost complete resolution of the infiltrates of the lobe auriculae.

Case 3

Figure S3

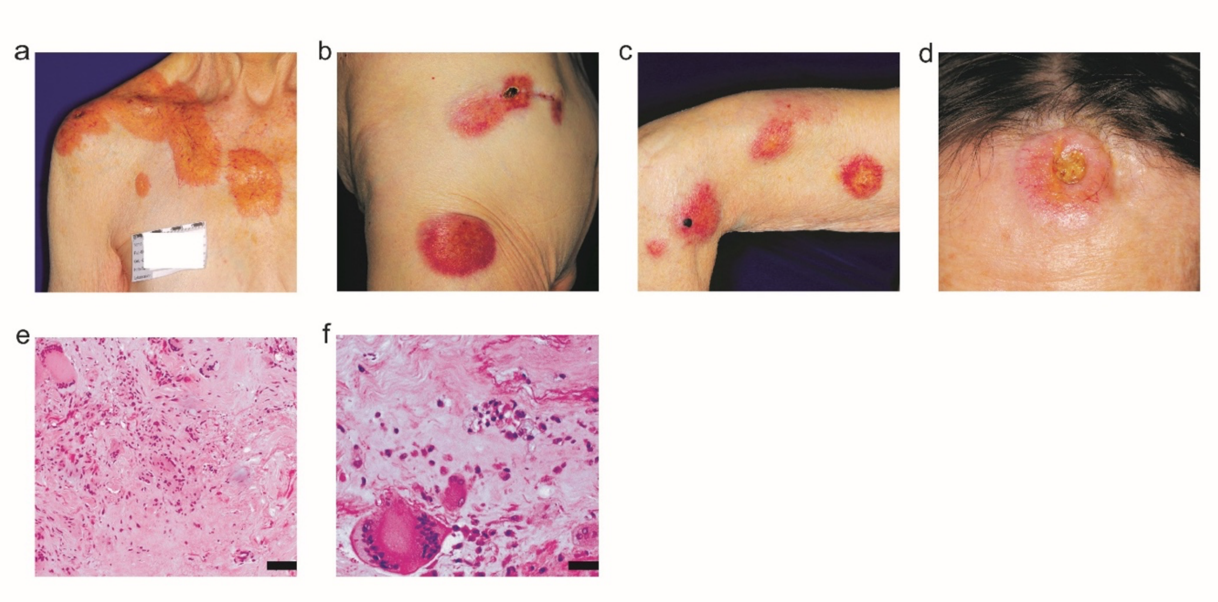

Figure S3: A-D, clinical images; E/F, histology (H&E, bar = 200 µm) showing necrobiosis and giant cells.

Clinical Presentation

A 72-years old patient suffered from skin changes that had existed for more than four years. In July 2014, a necrobiotic, pallisade-forming granuloma was diagnosed for the first time. The lesions were found cutaneous, subcutaneous and also muscular distributed over almost the entire body. In addition, there was an osseous involvement, a mediastinal and hilar lymph node involvement on both sides, as well as involvement in the lung area on both sides. The laboratory tests showed a decreased leukocyte and erythrocyte count. An increased gamma globulin fraction was confirmed in the protein electrophoresis. The alpha-1-globulin and alpha-2-globulin fractions were also increased. Albumin was lowered. The soluble inteleukin-2 receptor was significantly increased. Histomorphologically, there was no evidence of an underlying hematological disease.

Histological findings

The samples examined showed typical characteristics of NXG. The epidermis was mostly missing, in the area of the dermis numerous giant cells of the Touton type and Langerhans type

were visible. There were also numerous plasma cells. Cholesterol cracks or extensive necrobiosis were not present.

Therapy

Therapy with melphalan took place from November 2015 to January 2016. However, this therapy was discontinued when the disease progressed. The therapy was switched to lenalidomide in combination with dexamethasone and the disease showed a worsening. After discontinuing therapy with lenalidomide, high-dose corticosteroid therapy with 40 mg prednisolone per day was initiated. The ulcers were treated with Iruxol ointment and we used Neribas cream for basic care. In addition, therapy was started with Dapsone 50 mg daily. The dose was increased to 100 mg after one week and to 150 mg daily after another week.

Outcome

Therapy with Dapsone in connection with corticosteroids showed a partial response. Individual skin lesions decreased in size during treatment, but other lesions remained unchanged.

Case 4

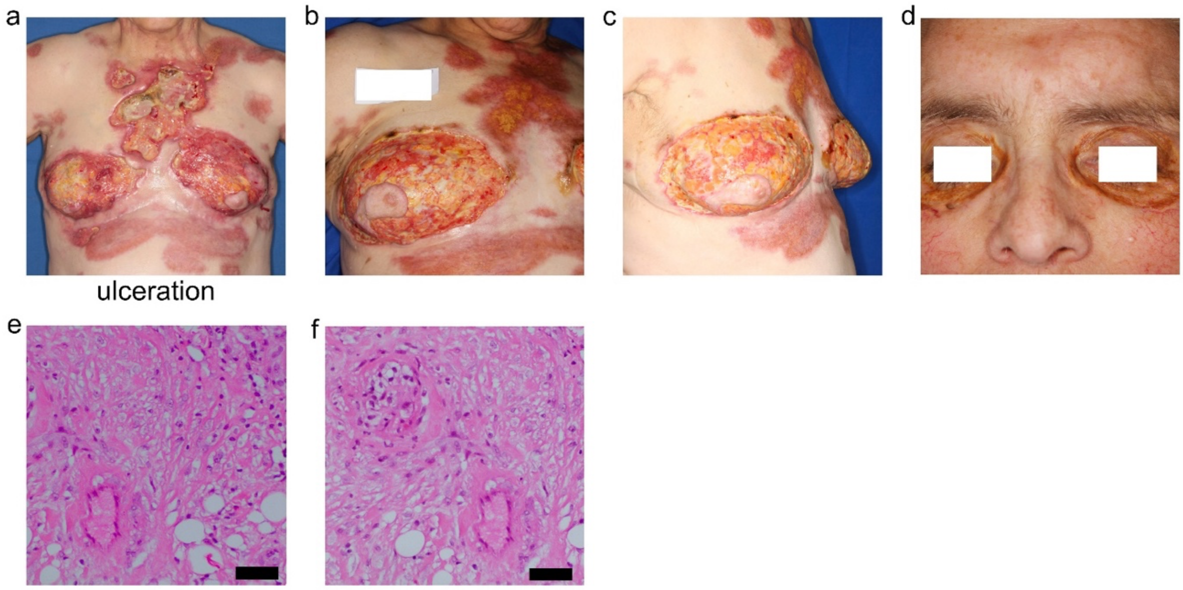

Figure S4: A-D, clinical images; E/F, histology (H&E, bar = 200 µm) showing giant cells and foamy histiocytes.

Clinical Presentation

A 69-year-old female presented with a 11-year history of worsening necrobiotic xanthogranuloma with dermal, ocular, vestibular and cochlear involvement. The olfactory system was also involved. The clinical examinationheavily fibrin-coated, deep ulceration with a livid border. In addition, the patient is also known to have monoclonal gammopathy IgG type kappa.

Histological findings

Pronounced fibrosis and erythrocyte extracts became visible in the central area of the preparation. In addition, foreign body giant cells could be seen, some of which were of the Touton type. There was no clear necrobiosis. In addition, CD68 staining took place. Numerous CD68 positive cells were found here.

Therapy

Therapy with Lenalidomide, was started previously, but had to be stopped because of pronounced leukocytosis. During the inpatient stay, we increased the corticosteroid dose to 80 mg per day. Lenalidomide therapy could be restarted in a reduced dose (5mg/die) and could be

applied alongside glucocorticoid therapy. In addition to steroid therapy, Ampho Moronal Suspension, Calcilac chewable tablets and Pantozol 40mg was added.

Outcome

There was a partial response to therapy with lenalidomide in combination with corticosteroids.

Case 5

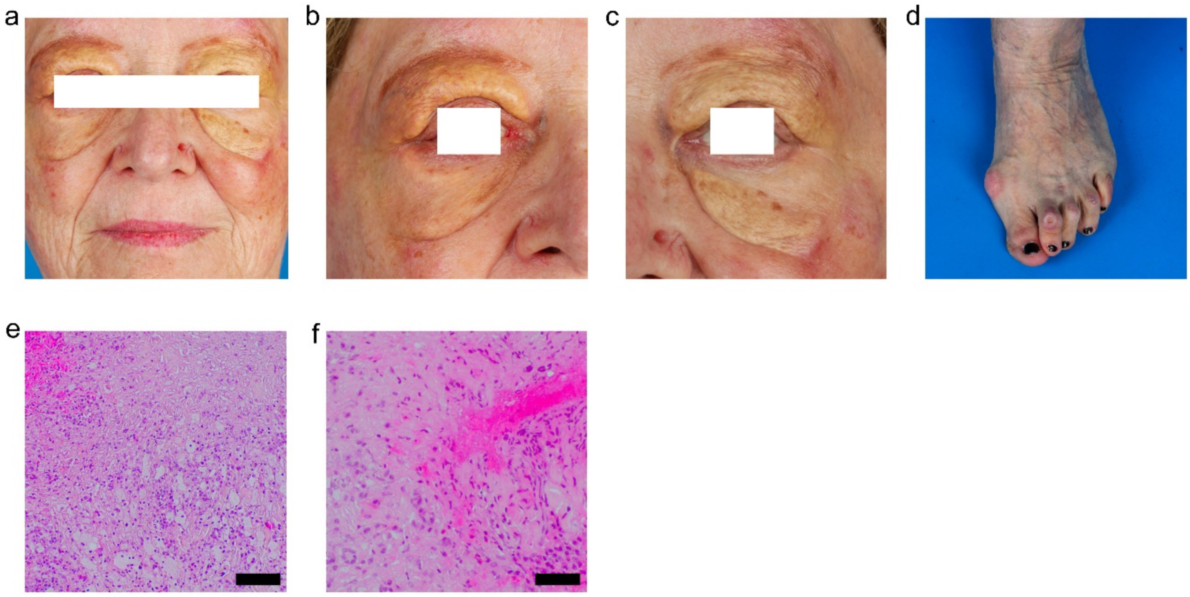

Figure S5: A-D, clinical images; E/F, histology (H&E, bar = 200 µm)

Clinical Presentation

A 67-year-old woman presented with periorbital swellings. The periorbital swellings have existed for about 10 years. The clinical examination showed a massive yellowish swelling in the area of both eyes (upper and lower eyelid). The rest of the integument appeared inconspicuous. Laboratory chemistry showed an increase in the L-chains of the lambda type and the free chains of the kappa type. There was also a slight eosinophilia, an increase in total IgE and monocytosis. There was no evidence of multiple myeloma or a T-cell lymphoma. Previous illnesses included hypothereosis, diabetes mellitus, and medically treated arterial hypertension. With regard to dermatological diseases, rosacea is present.

Histological findings

Biopsies showed that the epidermis was slightly papillomatous, but otherwise largely normal. Fibrosis and mixed-cell inflammatory infiltrates were found in the upper corium. The entire deep corium down to the subcutaneous tissue is interspersed by a very dense, mixed-cell and inflammatory infiltrate. There are many polynuclear histiocytes that form in granulomatous aggregates around a zone of necrobiosis. In the infiltrate there were also a lot of foam cells and isolated giant cells of the foreign body type. Cholesterol columns were not clearly detectable. PAS positive granules are found in the large foamy giant cells. In the mucin staining, however,

there was no evidence of mucin. Elastic fibers are missing in the entire inflammatory changed area.

Therapy

We started a high-dose dexamethasone therapy. As concomitant medication, we administered pantoprazole once a day, Calcilac chewable tablets twice a day and Ampho Moronal Suspension four times a day. Systemic glucocorticoid therapy was tapered slowly until the Cushing threshold dose (5 mg daily) was reached. The daily dose of 5mg (1-0-0) was maintained.

Outcome

The monotherapy therapy with glucocorticoids showed a good therapeutic response. All skin lesions showed regression.

Supplementary References

1. Wood, A.J., et al., *Necrobiotic xanthogranuloma: a review of 17 cases with emphasis on clinical and pathologic correlation.* Arch Dermatol, 2009. **145**(3): p. 279-84.
2. Olson, R.M., et al., *Periorbital Necrobiotic Xanthogranuloma Successfully Treated with Intravenous Immunoglobulin.* Case Rep Ophthalmol, 2018. **9**(1): p. 70-75.
3. Rose, G.E., et al., *Orbital xanthogranuloma in adults.* Br J Ophthalmol, 1991. **75**(11): p. 680-4.
4. Miszkiel, K.A., et al., *Radiological and clinicopathological features of orbital xanthogranuloma.* Br J Ophthalmol, 2000. **84**(3): p. 251-8.
5. Szalat, R., et al., *Physiopathology of necrobiotic xanthogranuloma with monoclonal gammopathy.* J Intern Med, 2014. **276**(3): p. 269-84.
6. Flann, S., et al., *Necrobiotic xanthogranuloma with paraproteinaemia.* Clin Exp Dermatol, 2006. **31**(2): p. 248-51.
7. Gun, D., Z. Demircay, and C. Demirkesen, *Necrobiotic xanthogranuloma in a burn scar.* Int J Dermatol, 2004. **43**(4): p. 293-5.
8. Kossard, S., et al., *Lipid and giant cell poor necrobiotic xanthogranuloma.* J Cutan Pathol, 2000. **27**(7): p. 374-8.
9. Szalat, R., et al., *Pathogenesis and treatment of xanthomatosis associated with monoclonal gammopathy.* Blood, 2011. **118**(14): p. 3777-84.
10. Kossard, S. and R.K. Winkelmann, *Necrobiotic xanthogranuloma.* Australas J Dermatol, 1980. **21**(2): p. 85-8.
11. Umbert, I. and R.K. Winkelmann, *Necrobiotic xanthogranuloma with cardiac involvement.* Br J Dermatol, 1995. **133**(3): p. 438-43.
12. Hunter, L. and A.F. Burry, *Necrobiotic xanthogranuloma: a systemic disease with paraproteinemia.* Pathology, 1985. **17**(3): p. 533-6.
13. Frank, S.B. and A.I. Weidman, *Xanthoma disseminatum; an unusual form with extension of xanthomatous changes into muscle.* AMA Arch Derm Syphilol, 1952. **65**(1): p. 88-94.
14. Novak, P.M., T.O. Robbins, and R.K. Winkelmann, *Necrobiotic xanthogranuloma with myocardial lesions and nodular transformation of the liver.* Hum Pathol, 1992. **23**(2): p. 195-6.
15. Winkelmann, R.K., et al., *Giant cell granulomatous pulmonary and myocardial lesions in necrobiotic xanthogranuloma with paraproteinemia.* Mayo Clin Proc, 1997. **72**(11): p. 1028-33.
